# Angiopoietin-2 attenuates angiotensin II-induced aortic aneurysm and atherosclerosis in apolipoprotein E-deficient mice

**DOI:** 10.1038/srep35190

**Published:** 2016-10-21

**Authors:** Hongyou Yu, Corey S. Moran, Alexandra F. Trollope, Lynn Woodward, Robert Kinobe, Catherine M. Rush, Jonathan Golledge

**Affiliations:** 1 Queensland Research Centre for Peripheral Vascular Disease, College of Medicine and Dentistry, James Cook University, Townsville, 4811, Australia; 2 Discipline of Anatomy, College of Medicine and Dentistry, James Cook University, Townsville, 4811, Australia; 3 Discipline of Biomedicine, College of Public Health, Medical and Veterinary Sciences, James Cook University, Townsville, 4811, Australia; 4 Department of Vascular and Endovascular Surgery, The Townsville Hospital, Townsville, 4814, Australia

## Abstract

Angiogenesis and inflammation are implicated in aortic aneurysm and atherosclerosis and regulated by angiopoietin-2 (Angpt2). The effect of Angpt2 administration on experimental aortic aneurysm and atherosclerosis was examined. Six-month-old male apolipoprotein E deficient (ApoE^−/−^) mice were infused with angiotensin II (AngII) and administered subcutaneous human Fc-protein (control) or recombinant Angpt2 (rAngpt2) over 14 days. Administration of rAngpt2 significantly inhibited AngII-induced aortic dilatation and rupture of the suprarenal aorta (SRA), and development of atherosclerosis within the aortic arch. These effects were blood pressure and plasma lipoprotein independent and associated with Tie2 activation and down-regulation of monocyte chemotactic protein-1 (MCP-1) within the SRA. Plasma concentrations of MCP-1 and interleukin-6 were significantly lower in mice receiving rAngpt2. Immunostaining for the monocyte/macrophage marker MOMA-2 and the angiogenesis marker CD31 within the SRA were less in mice receiving rAngpt2 than controls. The percentage of inflammatory (Ly6C^hi^) monocytes within the bone marrow was increased while that in peripheral blood was decreased by rAngpt2 administration. In conclusion, administration of rAngpt2 attenuated angiotensin II-induced aortic aneurysm and atherosclerosis in ApoE^−/−^ mice associated with reduced aortic inflammation and angiogenesis. Up-regulation of Angpt2 may have potential therapeutic value in patients with aortic aneurysm and atherosclerosis.

Abdominal aortic aneurysm (AAA) is an abnormal dilatation of the abdominal aorta affecting 2–5% of men aged over 65 years that can lead to life-threatening aortic rupture^1^. Intensive investigation over the last two decades has not yet led to discovery of a drug-based therapy to limit AAA growth^2^. The pathogenesis of AAA is a complex process involving inflammation, extracellular matrix remodelling and angiogenesis^1^. Atherosclerosis is a usual finding in biopsies of end-stage AAA. Athero-thrombosis is also a recognised risk factor for AAA^1^. Nevertheless, it is still unknown whether atherosclerosis is causative for AAA or whether the association simply reflects common risk factors^3^,^4^.

Inflammation and extracellular matrix degradation provide a pro-angiogenic environment conducive to neo-vessel formation^5^. Angiogenesis is believed to promote AAA and atherosclerosis development and progression through encouraging extracellular matrix remodelling and facilitating inflammatory cell infiltration^1^,^6^,^7^. AAA rupture is associated with neovascularization^8^. A higher density of micro-vessels has been reported in biopsies from ruptured than intact AAAs^9^.

Angiopoietin-2 (Angpt2) is a well-studied member of a family of vascular growth factors known to regulate angiogenesis^10^. Angpt2 was originally identified by virtue of its ability to interfere with the action of angiopoietin-1 through competition for the receptor Tie2^11^. Transgenic mice over expressing Angpt2 have disrupted vessel formation that is similar but more severe than mice with deficiency of angiopoietin-1 or Tie2^12^. In humans, increased serum Angpt2 is associated with AAA prevalence and cardiovascular mortality in older men^13^, however, a direct role for Angpt2 in AAA remains unclear.

Given the significance of angiogenesis and inflammation in AAA and atherosclerosis, we hypothesised that Angpt2 would be important in the development of AAA and atherosclerosis and examined this in the angiotensin II (AngII)-infused Apolipoprotein E deficient (ApoE^−/−^) mouse model.

## Results

### Administration of rAngpt2 reduced aortic aneurysm development in response to AngII infusion

**Figure 1:**
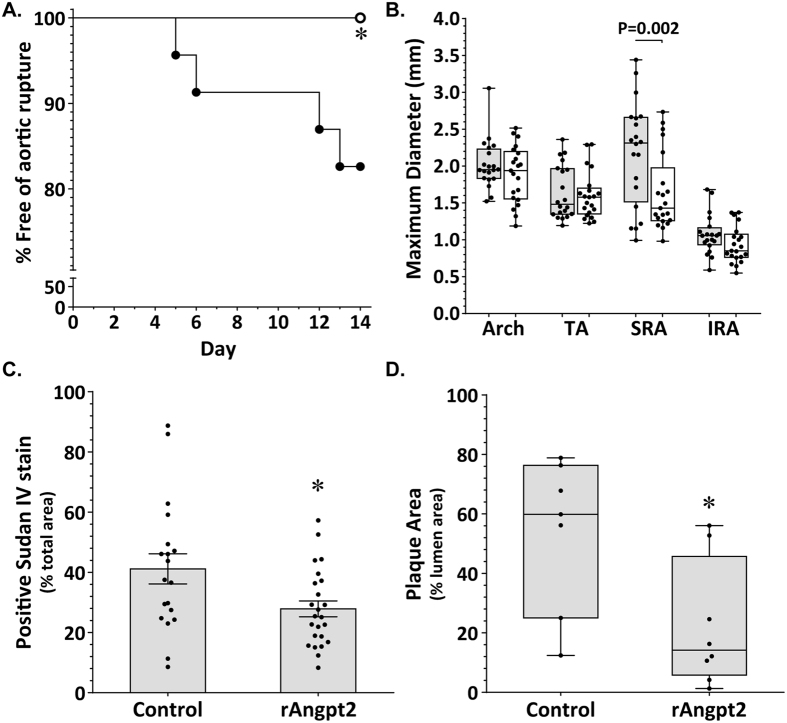
Effect of rAngpt2 on AngII-induced aortic dilatation and atherosclerosis in *ApoE*^−/−^mice. (**A**) Kaplan-Meier curves of survival free of aneurysm rupture in AngII-infused ApoE^−/−^ mice administered rAngpt2 (○; 4.0 mg/kg/48 hrs, s.c.) or control peptide (•); *P = 0.034 compared to controls using Log-rank (Mantel-Cox) test. (**B**) Regional aortic diameters for AngII-infused ApoE^−/−^ mice administered rAngpt2 (white; 4.0 mg/kg/48 hrs, s.c.; n = 24) or control peptide (grey; n = 23) determined by morphometry at sacrifice (day 14). Data expressed as median and interquartile range with maximum and minimum data points (whiskers) for maximum diameters (mm) and compared by Mann-Whitney U test. *TA, thoracic aorta; SRA, suprarenal aorta; IRA, infrarenal aorta*. (**C**) Sudan IV staining area within the aortic arch of mice receiving rAngpt2 (4.0 mg/kg/48 hrs, s.c.; n = 24) compared to controls (n = 19). Data expressed as mean ± SEM for positive staining area relative to total specimen area (%); *P = 0.017 compared by unpaired-*t* test. (**D**) Atherosclerotic plaque within the brachiocephalic artery (BCA) of mice receiving rAngpt2 (4.0 mg/kg/48 hrs, s.c.; n = 8) compared to controls (n = 7). Data expressed as median and interquartile range with maximum and minimum data points (whiskers) for plaque cross-sectional area relative to total luminal area (%); *P = 0.009 compared by Mann-Whitney U test.

A total of 50 6-month-old male ApoE^−/−^ mice were infused with angiotensin II (AngII) for 14 days. Mice received rAngpt2 (n = 25) or human Fc protein (control; n = 25) at a dose 4 mg/kg every other day commencing 24 hours prior to AngII infusion. Two mice in the control group and one mouse in the rAngpt2 group died during the experimental period of non-aneurysm related causes and were excluded from the study. Aortic rupture occurred in 4 out of the 23 control mice (17%) at days 5, 6, 12 and 13. There were no aortic ruptures in mice receiving rAngpt2 (P = 0.045, Fisher’s exact test). Accordingly, intervention improved survival from aortic rupture from 83% in control mice to 100% in mice receiving rAngpt2 (P = 0.034; Fig. 1A). The response to AngII infusion ranged from focal dilatations of the suprarenal aorta (SRA) to complex dissection throughout the vessel (see Supplementary Fig. S1). The severity of aortic response to AngII was scored using a scale based upon gross appearance as previously described (Table 1)^14^. Quantitative morphometric analysis of the aortas of the mice demonstrated significantly smaller mean maximum SRA diameter in mice receiving rAngpt2 than control (P = 0.002, Fig. 1B). Smaller diameters of the aortic arch, thoracic (TA) and infrarenal aorta (IRA) were also observed in mice receiving rAngpt2 although differences were not statistically significant compared to controls (Fig. 1B). rAngpt2 administration did not influence AngII-induced elevation of blood pressure (Table 2).

### Atherosclerosis within the aortic arch was reduced in mice administered rAngpt2

AngII infusion accelerates atherosclerosis development in ApoE^−/−^ mice particularly within the aortic arch^6^,^15^. Intimal atheroma within the aortic arch and plaque development within the brachiocephalic artery (BCA) of surviving mice was assessed by Sudan IV staining and Haematoxylin and Eosin histology, respectively. Mean positive Sudan IV staining area within the aortic arch was reduced from 41.15 ± 4.99% in control mice to 27.87 ± 2.63% in mice receiving rAngpt2 (P = 0.017; Fig. 1C; Supplementary Fig. S2). Similarly, the median cross-sectional plaque area within the BCA was reduced 3-fold in mice administered rAngpt2 compared to control (P = 0.009; Fig. 1D; Supplementary Fig. S3). Circulating lipid profile was similar in the intervention and control groups (Table 2).

### AngII-induced aortic inflammation was reduced in mice administered rAngpt2

**Figure 2:**
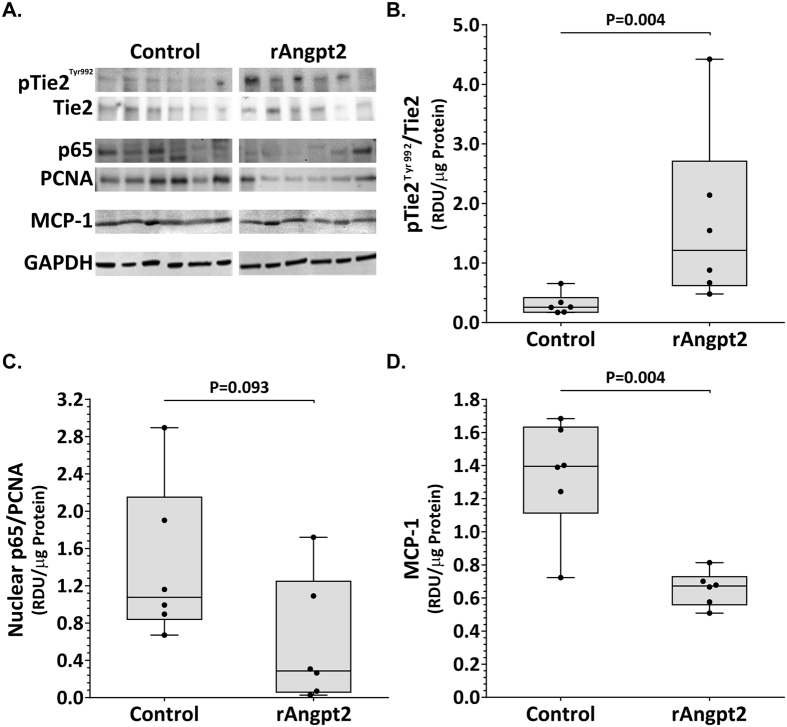
Effect of rAngpt2 on Tie2 phosphorylation and inflammation markers within the suprarenal aorta. (**A**) Representative western blots for Tie2/phosphorylated Tie2^Tyr992^, nuclear (NFκB) p65/PCNA, and MCP-1. Tie2^Tyr992^ phosphorylation (**B**), nuclear p65 (**C**), and MCP-1 (**D**) levels within the SRA of AngII-infused ApoE^−/−^ mice administered rAngpt2 (4.0 mg/kg/48 hrs, s.c.; n = 6) or controls (n = 6) after 14 days. Data expressed as median and interquartile range with maximum and minimum data points (whiskers) for relative density units (RDU) per microgram (μg) protein, compared by Mann-Whitney U test.

**Figure 3:**
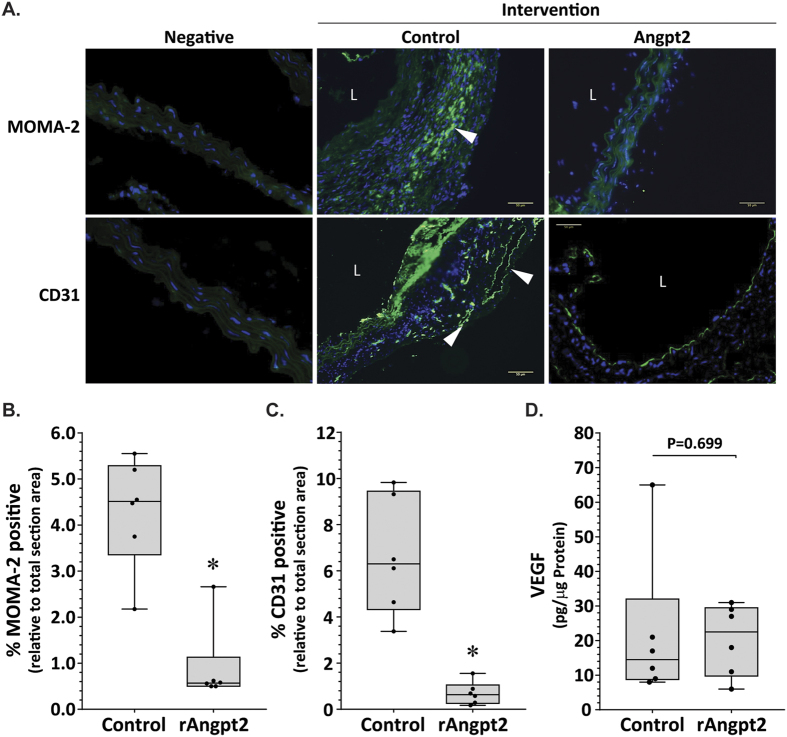
Effect of rAngpt2 on monocyte/macrophage infiltration and markers of angiogenesis within the suprarenal aorta. (**A**) Immunostaining for macrophages (arrow) and angiogenesis with MOMA-2 (upper panel) and CD31 (lower panel), respectively. Response to AngII after 14 days in SRA of ApoE^−/−^ mice administered rAngpt2 (4.0 mg/kg/48 hrs, s.c.; n = 6) or control (n = 6). *L, lumen;* cell nuclei stained blue; 200x magnification; scale bar, 50 μm; negative is isotype IgG control and represents background fluorescence. Data for MOMA-2 (**B**) and CD31 (**C**) expressed as median and interquartile range with maximum and minimum data points (whiskers) for positive staining area relative to total specimen area (%); *P < 0.01 compared by Mann-Whitney U test. (**D**) Concentration of VEGF within the SRA of mice receiving rAngpt2 (4.0 mg/kg/48 hrs, s.c.; n = 6) compared to controls (n = 6). Data expressed as median and interquartile range with maximum and minimum data points (whiskers) for picograms (pg) of VEGF per μg protein, compared by Mann-Whitney U test.

Angpt2 can sensitise endothelial cells to inflammatory stimuli through the regulation of Tie2^11^,^16^. Western blotting was used to determine phosphorylated Tie2/total Tie2, nuclear (NFκB) p65/PCNA, and MCP-1 protein levels within SRA tissue (Fig. 2A). Tie2 phosphorylation was up-regulated within the aortic tissue of mice receiving rAngpt2 (P = 0.004; Fig. 2B). Mice receiving rAngpt2 had lower SRA levels of nuclear p65 compared with controls, although the difference was not statistically significant (P = 0.093; Fig. 2C). Expression of monocyte chemotactic protein (MCP)-1 within SRA tissue was positively correlated with maximum SRA diameter (*r*^2^ = 0.781; P = 0.043). Median aortic MCP-1 concentration was markedly reduced in mice administered rAngpt2 compared to controls (P = 0.004; Fig. 2D). Immunostaining area for monocyte/macrophages (MOMA-2) in SRA sections was significantly reduced in mice administered rAngpt2 compared to controls (P = 0.004; Fig. 3A upper panel & 3B). Angiogenesis was assessed in SRA tissue obtained at the end of experiments by immunostaining for CD31 and ELISA for VEGF. Both intimal and adventitial micro-vessel endothelial cells were positively stained for CD31 (Fig. 3A lower panel). rAngpt2 administration was associated with a lower median CD31-positive staining area within the SRA adventitia (P = 0.002; Fig. 3C). Mice receiving rAngpt2 had similar median aortic VEGF concentration as controls (Fig. 3D).

### rAngpt2 administration affected circulating profile of Ly6C^hi^ monocytes

**Figure 4:**
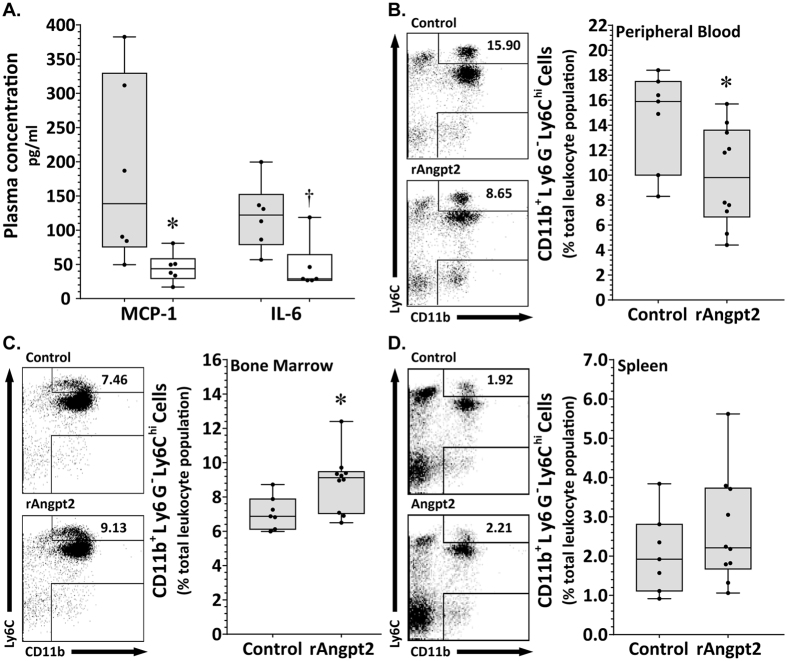
Effect of rAngpt2 on blood and tissue profile of Ly6C-positive inflammatory monocytes. (**A**) Circulating concentration of MCP-1 and IL-6 in AngII-infused mice receiving rAngpt2 (4.0 mg/kg/48 hrs, s.c.; n = 6) or control peptide (n = 6) after 14 days. Data expressed as median and interquartile range with maximum and minimum data points (whiskers) for picograms (pg) of protein per ml plasma; *P = 0.010 and ^†^P = 0.013 compared by Mann-Whitney U test. Inflammatory (Ly6C^hi^) monocytes within the circulation (**B**), bone marrow (**C**), and spleen (**D**) of AngII-infused mice receiving rAngpt2 (4.0 mg/kg/48 hrs, s.c.; n = 10) or control peptide (n = 7) after 14 days. Data expressed as median and interquartile range with maximum and minimum data points (whiskers) for CD11b^+^Ly6G^-^Ly6C^hi^ cells relative to total leukocyte population (%); *P = 0.019 (**B**,**C**) compared by Mann-Whitney U test.

At completion of the AngII infusion period, median plasma concentrations of MCP-1 and interleukin-6 were 3- and 4-fold lower, respectively, in mice receiving rAngpt2 compared to controls (Fig. 4A; P = 0.010 and 0.013, respectively). There was no significant difference between median plasma tumour necrosis factor concentrations in mice receiving rAngpt2 and those injected with control protein (3.68, 3.41–6.84 pg/ml versus 4.49, 3.21–8.34 pg/ml, respectively; P = 0.794, n = 6). IFN-γ and IL-12p50 were not detectable in the plasma of either group. Mice administered rAngpt2 had lower median levels of circulating inflammatory (CD11b^+^Ly6G^-^Ly6C^hi^) monocytes compared to control animals (P = 0.019; Fig. 4B), but a significantly higher median percentage of inflammatory monocytes within the bone marrow (P = 0.019; Fig. 4C). The median percentage of inflammatory monocytes in the spleen was similar in the two groups (P = 0.516; Fig. 4D). A very low mean percentage of the total circulating leukocyte population (~0.5%) in blood of mice prior to AngII infusion (baseline) was identified as Tie2-expressing (CD11b^+^Tie2^+^). The mean percentage of circulating CD11b^+^Tie2^+^ cells following 14 days of AngII infusion remained similar to baseline (P > 0.05; Supplementary Fig. S4).

## Discussion

This study investigated the effect of Angpt2 on AAA development and atherosclerosis in the ApoE^−/−^ mouse model. Systemic administration of rAngpt2 attenuated AngII-induced AAA formation and atherosclerosis progression. Examination of aortic tissue showed that aortic monocyte/macrophage infiltration, aortic concentration of MCP-1, and aortic neo-vascularisation was reduced in mice receiving rAngpt2. In addition, these mice exhibited lower percentage of circulating Ly6C^hi^ (inflammatory) monocytes that corresponded with reduced concentration of plasma MCP-1. We have reported previously that serum Angpt2 is elevated in men with AAA and associated with an increased risk of cardiovascular mortality in this population^13^, although whether this was cause or consequence remained unclear. Here we provide *in vivo* evidence supporting a protective role for Angpt2 in experimental AAA and atherosclerosis.

Angiopoietin-Tie signalling is an important regulator of vascular homeostasis, angiogenesis, and inflammation^10^,^17^. Tie2 is activated in order to maintain the quiescent state of the vasculature^14^ whereas Tie2 is deactivated in the process of angiogenesis and inflammation^16^,^18^,^19^. Therefore, Tie2 activation is important in maintaining the integrity of the endothelium of the aorta. Angpt2, unlike angiopoietin-1, has a complex role and dynamically regulates Tie2 in a context dependent manner^20^,^21^. Angpt2 is mainly expressed by endothelial cells and stored in intracellular granules that can be released rapidly to de-activate Tie2 in an autocrine manner in response to inflammatory and angiogenic stimuli^21^. In contrast exogenously administered Angpt2 has been demonstrated to activate Tie2 and limit angiogenesis both *in vivo* and *in vitro*^20^,^22^,^23^,^24^,^25^. Moreover, it has been reported that exogenous Angpt2 administration inhibited leukocyte infiltration in the presence of inflammatory stimuli^22^,^26^. In the current study, we report that exogenous rAngpt2 administration induced Tie2 activation and reduced monocyte/macrophage infiltration within the aortic wall in the presence of AngII, an important regulator of the inflammatory response^27^.

Angpt2 has critical roles in angiogenesis. It has been reported that up-regulation of Angpt2 in animal models of cancer disrupted tumour vasculature^10^,^23^,^24^,^25^. In the current study we found that mice receiving systemic administration of rAngpt2 had lower aortic staining for the angiogenesis marker CD31 than controls. There is evidence that AAA and atherosclerosis are associated with excessive angiogenesis in both patients and animal models^8^,^9^,^28^,^29^. Indeed, anti-angiogenic interventions have been shown to attenuate experimental AAA^28^,^30^. Prolonged exposure of endothelial cells to Angpt2 induces apoptosis and vessel regression in the absence of VEGF^12^,^31^,^32^. In line with this, we found that reduced AAA development in mice administered Angpt2 was associated with decreased aortic expression of CD31, with no change in aortic tissue level of VEGF. Tie2-expressing monocytes/macrophages have been shown to be proangiogenic in some animal models^33^,^34^. However, it is not likely that Tie2-expressing monocytes/macrophages play an important role in the findings of the current study since mice receiving rAngpt2 had less micro-vessel formation within the aorta. Moreover, Tie-2-expressing monocytes/macrophages were barely detectable in blood of ApoE^−/−^ mice prior to (baseline) and after the AngII infusion period.

The extent of angiogenesis correlates with the severity of inflammatory cell infiltration in AAA biopsies^9^,^35^. AngII induced AAA and atherosclerosis have been associated with aortic macrophage accumulation^6^,^36^. Such aortic inflammation may in part be a consequence of AngII induced angiogenesis since newly formed pathological capillaries are normally poorly organised and highly permeable^37^. Furthermore, infusion of AngII promotes NF-κB activation and up-regulation of aortic MCP-1 expression, which are effects that are closely associated with aortic aneurysm progression and rupture^27^,^38^,^39^,^40^,^41^. In addition to our observation that rAngpt2 administration reduced markers of angiogenesis we also demonstrated less aortic macrophage staining and MCP-1 expression in mice that received rAngpt2. Maximum aortic diameter is commonly presented as the primary outcome measure in experimental AAA studies^36^,^42^,^43^. AAA diameter is an important clinical indicator of rupture risk and routinely used in selecting patients for surgical intervention^44^. Ultimately any medical treatment for aortic aneurysm aims to limit AAA rupture. We utilised the AngII-infused *ApoE*^−/−^ mouse model of aortic aneurysm in which aortic dilatation results from breaks in medial elastic lamellae, bleeding into the artery wall and aortic wall inflammation, a process that leads to acute aortic rupture in approximately 30% of mice^36^,^45^. This model allowed the assessment of both survival free from aortic rupture and maximum aortic diameter. Administration of Angpt2 limited AngII-induced aortic dilatation and protected against aortic rupture.

Ly6C^hi^ inflammatory monocytes express C-C chemokine receptor type 2 (CCR2), a receptor for MCP-1. Markedly reduced aortic dilatation^46^ and plaque formation^47^ in CCR2-deficient mice suggests the importance of CCR2-MCP-1 signalling in AAA and atherosclerosis development in rodent models. CCR2-MCP-1 signalling controls Ly6C^hi^ inflammatory monocyte egress from the bone marrow to the circulating blood and recruitment to inflammatory sites, where they differentiate into activated macrophages^39^. Inflammatory monocytes have been implicated in the pathogenesis of AAA and atherosclerosis^48^,^49^,^50^. We found less inflammatory monocytes within the circulation of mice receiving rAngpt2 at day 14 of our experiment when the plasma concentration of MCP-1 was significantly less than control mice. We also found a higher percentage of Ly6C^hi^ cells within the bone marrow of mice receiving rAngpt2. These findings suggested that rAngpt2 administration restricted inflammatory monocyte egress from the bone marrow.

The effect of Angpt2 on AAA and atherosclerosis has not previously been investigated in the AngII-infused AAA mouse model. We used systemic administration of rAngpt2 which is relevant to situations where angiogenesis is being therapeutically targeted by intravenous agents^51^. In a recent clinical trial, for instance, the anti-angiogenic agent bevacizumab, a humanized monoclonal antibody neutralising VEGF activity, was administered intravenously in order to limit cancer progression^54^. Our results are consistent with other studies in which systemic Angpt2 administration has been shown to stimulate regression of neovascularisation^10^,^23^,^24^ and a previous study in which up-regulation of Angpt2 by systemic administration of an adenovirus vector inhibited atherosclerosis progression by 40%^52^.

The current study has a number of limitations. Firstly, rAngpt2 administration was associated with reduction in both AAA and atherosclerosis. Thus it is possible that the reduction in atherosclerosis may have caused a secondary reduction in AAA. The role of atherosclerosis in AAA is controversial and it is not likely that atherosclerosis is the primary cause of AAA in the model used in the current study thus we think this is unlikely^4^. Secondly, since Angpt2 has many actions the precise mechanism by which rAngpt2 limited AAA and atherosclerosis in our study is not certain. Angpt2 is needed for functional lymphatics as mice deficient in Angpt2 show defective lymphatic patterning^53^. However, we did not assess lymphatics in the current study.

In summary, the current study suggests that rAngpt2 administration limits AAA and atherosclerosis in a mouse model associated with reduced angiogenesis and inflammation. These results suggest the potential beneficial functions of rAngpt2 administration in cardiovascular disease and provide a possible strategy for AAA prevention and treatment.

## Methods

### Recombinant angiopoietin-2 and control human Fc protein

Recombinant human angiopoietin-2 (rAngpt2) and control human Fc protein were kindly provided by Regeneron Pharmaceuticals (Tarrytown, NY, USA). The rAngpt2 used in this study is equivalent to AngF2-Fc-F2 described in detail in previous publications^54^.

### Mouse studies

The use of animals for this work conformed to the Guide for the Care and Use of Laboratory Animals (National Institutes of Health, USA). Approval for the mouse studies and experimental work performed was obtained from and in accordance with the James Cook University Animal Ethics Committee (A1671). Six month old male ApoE^−/−^ mice were obtained from the Animal Resources Centre (Canning Vale, Australia). Mice were housed in an individually-ventilated, temperature/humidity-controlled cage system (Aero IVC Green Line; Tecniplast) on a 12-hour light/dark cycle, and maintained on normal laboratory chow and water ad libitum. ApoE^−/−^ mice were allocated to receive rAngpt2 or control peptide. The planned primary outcome measure was SRA diameter, as this is the main site of aneurysm formation in the mouse model employed^6^,^36^. Based on a previous study it was estimated that a sample size of 20 mice per group would have at least 80% power to detect a 25% difference in SRA diameter (alpha 0.05)^55^. In order to allow for potential dropouts due to mice fatalities we included 25 mice in each group. All of these mice were also used to assess survival free from aneurysm rupture and atherosclerosis lesion area in the aortic arch. The other secondary outcomes in this study were assessed in one subset experiment (n = 10 in each group) and included blood pressure; plasma lipids; aortic and plasma concentration of pro-inflammatory cytokines; peripheral blood, bone marrow and spleen levels of inflammatory monocytes; monocyte/macrophage infiltration of the aortic wall; and angiogenesis within the aortic wall.

### Angiotensin II induced AAA model and administration of rAngpt2

All mice received 1.44 mg/kg/day of AngII (Sigma-Aldrich) via subcutaneous osmotic mini-pumps (Model 1004, ALZET) for 14 days as previously described^56^. In brief, mice were anaesthetized by intra-peritoneal injection of ketamine (150 mg/kg) and xylazine (10 mg/kg), and then osmotic mini-pumps containing AngII dissolved in sterile water were inserted into the subcutaneous space along the left dorsal midline. rAngpt2 or control peptide-Fc fusion protein was administered subcutaneously every other day at a dose of 4 mg/kg starting the day before AngII infusion commenced.

### Tissue collection

Mice were euthanised by CO_2_ asphyxiation at the completion of the experiment. The aorta was exposed under a dissection microscope (Leica). Blood was collected into lithium heparin coated tubes (BD Bioscience) from the right ventricle and centrifuged to collect plasma that was stored at −80 °C for future assessment. The aorta was carefully flushed with phosphate buffered saline (PBS) through the left ventricle and harvested free from fat and connective tissue. The whole dissected aorta was digitally photographed (Coolpix 4500, Nikon) on a graduated template^44^. Then aortic tissues were snap frozen in Optimal Cutting Temperature compound and stored at −80 °C for future analysis. Mice that died prior to completion of the experimental period were subject to post-mortem and aortas were harvested for subsequent aortic diameter measurement. Rupture was confirmed by intra-thoracic, intra-abdominal or retro-peritoneal blood.

### Measurement of aortic diameters

Maximum diameters of the following aortic regions were measured in harvested aortas using established techniques: aortic arch (from aortic valve to left subclavian artery), thoracic aorta (from the end of the arch to the diaphragm), SRA (from the diaphragm to the renal arteries), and infrarenal aorta (from the renal arteries to bifurcation of the iliac arteries). Maximum diameter of each aortic region was determined using Adobe Photoshop CS5 software (V12, Adobe) as previously described^43^. We have previously reported good inter-observer reproducibility of these measurements^43^,^57^.

### Assessment of atherosclerosis lesion area in the aortic arch

All aortic arches from the mice surviving to the end of the experiments were used to evaluate the area of intimal atherosclerosis using Sudan IV staining as previously described^43^. In brief, aortic arches were opened longitudinally to expose the intima and pinned on wax. Tissues were fixed in 75% ethanol for 15 min followed by staining using Sudan IV solution (0.1% Sudan IV dissolved in 1:1 v/v acetone and 70% ethanol) for 10 min. Excess staining solution was removed by a 15 min 80% ethanol wash. The stained arch tissues were digitally photographed on a graduated template and the Sudan IV staining area was measured using Adobe Photoshop CS5 software (V12, Adobe) as previously described^55^. We have previously reported good inter-observer reproducibility of these measurements^43^,^55^.

### Non-invasive assessment of blood pressure using a tail-cuff system

Blood pressure was measured using a computerised non-invasive tail-cuff system (Kent Scientific, USA) at day 0 (baseline) and day 14 (week 2). Mice were acclimatized to the device before measurements. We have previously reported good inter-observer reproducibility of these measurements^56^.

### Plasma lipids measurement

Plasma samples, snap frozen and stored at −80 °C, were used to measure circulating lipids using an Abcam high density lipoprotein (HDL) and low and very low density lipoprotein (LDL/VLDL) Cholesterol Assay Kit (Abcam) following the manufacturer’s instructions. Briefly, plasma was precipitated using the provided precipitation buffer followed by centrifugation. The HDL fraction was precipitated while low and LDL/VLDL fraction remained in the supernatant. The concentration of cholesterol in each fraction was measured using a cholesterol esterase reagent in the reaction buffer. A standard curve was created using the provided cholesterol standard. The optical density (OD) value was measured at 570 nm using a micro plate reader (BMG Labtech). The concentration of cholesterol in each fraction was determined according to the standard curve.

### Western blotting

Six SRA tissues were randomly chosen from the rAngpt2 and control groups to assess expression of the pro-inflammatory cytokine MCP-1 by Western blotting. Tissues were homogenized by three freeze-thaw cycles. Total protein was extracted by sonication with 3–4 bursts of 10 seconds each in radio-immunoprecipitation assay (RIPA) buffer (50 mM Tris-HCl PH 7.4, 150 mM NaCl; 2 mM EDTA, 1% TritonX-100, 0.1% SDS, and 0.1% sodium deoxycholate) in the presence of a protease inhibitor cocktail and phosphatase inhibitor (Roche). Nuclear protein was extracted using the NE-PER nuclear protein extraction kit (Piercenet). Protein concentration was determined using a Bradford protein assay kit (BioRad). Samples (30 μg of protein per lane) were loaded onto 4–20% Mini-PROTEAN TGX precast polyacrylamide gels (Bio-Rad) and were then separated at 110 V for 70 min. Separated proteins were transferred to a polyvinylidene fluoride membrane (BioRad). Non-specific antibody sites were blocked with 5% bovine serum albumin (BSA) for 60 min at room temperature. Membranes were probed with primary rat monoclonal anti-monocyte chemotactic protein-1 (MCP-1; SantaCruz), rabbit polyclonal anti-p65 (Abcam), rabbit polyclonal anti-proliferating cell nuclear antigen (PCNA; SantaCruz), mouse monoclonal anti-Tie2 (clone Ab33; Millipore), rabbit polyclonal anti-phosphorylated Tie2 (pTie2^Tyr992^ ; Cell Signaling), or rabbit monoclonal anti-GAPDH (cell Signaling) at a 1:1000 dilution in 2% BSA at 4 °C overnight. Anti-rabbit, mouse or rat HRP conjugated IgG (1:2500) (Dako Cytomation) was used as a secondary antibody. Blots were developed with Clarity Western ECL Substrate (BioRad) and Image Lab software (V5, Bio-rad) was used for densitometry quantification. The data were expressed as relative fold of control by normalising the relevant protein’s band intensity to the mean band intensity of the control mice.

### Enzyme-linked immunosorbent assay (ELISA)

The mouse VEGF Quantikine ELISA kit (R&D) was used to assess VEGF protein concentrations in aortic tissue according to the manufacturer’s instructions. In brief, 30 μg of protein from the aortic tissue was added to a pre-coated 96-well-plate and incubated at room temperature for 2 hours followed by five washes with wash buffer. A polyclonal antibody against mouse VEGF conjugated to horseradish peroxidase was added to each well and incubated at room temperature for 2 hours. Then the substrate solution was added to each well to incubate at room temperature for 30 min followed by adding stop solution. A standard curve was created using the provided standard. The OD value was measured at 450 nm using a micro plate reader (BMG Labtech). The concentration of VEGF in each sample was determined according to the standard curve.

### Flow cytometry

At the end of the experiments, peripheral blood was collected via cardiac puncture into lithium heparin coated tubes (BD Bioscience). Bone marrow cells were harvested by inserting a 25 gauge needle into the left femur and injecting 500 μl of Dulbecco’s Phosphate Buffered Saline without Ca^2+^ and Mg^2+^ (DPBS, Gibco) containing 0.2% BSA and 1 mM EDTA (Amresco). Spleens were homogenized in DPBS containing 0.2% BSA and 1 mM EDTA and filtered through 60 micron nylon filters. Typically 100 μl of peripheral blood and 10^6^ cells from bone marrow or spleen were used for flow cytometry. Cell surface staining for flow cytometry was conducted using standard procedures^18^. The following monoclonal antibodies were used: anti-CD11b (M1/70)-APC, anti-Ly6G (1A8)-PerCP-Cy5.5, anti-Ly6C (AL-21)-FITC (all from BD pharmingen); anti-Tie2 (TEK4)-PE (eBioscience). Briefly, erythrocytes were depleted using red blood cell (RBC) lysis buffer (BioLegend) before cell suspensions were pre-incubated for 20 min with anti-CD16/32 to prevent the Fc receptors from binding antibodies. Cell suspensions were incubated with staining antibody cocktails for 1 hour at room temperature in the dark. Viable leukocytes were identified by forward/side scatter profile and fluorescence-minus-one (FMO) controls were used to determine positive and negative boundaries. Inflammatory monocytes were defined as CD11b^+^Ly6G^−^Ly6C^hi^. Flow cytometry was performed on a FACSCalibur (BD bioscience) and data were analysed with BD CellQuest pro software (v5.1 BD bioscience). Data were expressed as percentage of inflammatory monocytes in total live leukocyte populations.

### Assessment of plasma pro-inflammatory cytokines

Cytometric bead array assays (BD Bioscience) were used to assess plasma interleukin (IL)-6, IL-12p70, tumour necrosis factor, interferon gamma, and MCP-1 concentrations according to the manufacturer’s instructions. In brief, 50 μl of plasma per sample was mixed with 50 μl APC-conjugated beads and 50 μl PE detection reagent. The mixtures were incubated at room temperature for 2 hours in the dark. Then samples were washed with washing buffer and centrifuged at 250 g for 5 min. The beads were resuspended in 300 μl washing buffer and were assessed immediately with the Cyan-ADP analyser (Beckman Coulter). Cytokine concentrations were determined from a standard curve using the standards provided. Data were analysed with FCAP Array Analysis Software (v3, Soft Flow, Inc.).

### Immunofluorescence staining and quantification of monocytes/macrophage and angiogenesis within the aortic wall

Six SRAs were randomly chosen from each group for immunofluorescence staining. 6 μm cryostat sections were cut for immunofluorescence staining. Briefly, sections were fixed in cold acetone at −20 °C for 20 min and then blocked with 5% normal goat serum in PBS containing 0.5% BSA and 0.1% Triton X-100 for 60 min at room temperature. Sections were incubated with rat anti-MOMA-2 (Abcam, ab33451) or rat anti-CD31 (MEC 13.3; Santa Cruz, sc-18916) antibodies at a dilution of 1:100 in PBS containing 0.5% BSA overnight at 4 °C. Rat IgG (Sigma I4131) was used as isotype control. After washing, samples were incubated with Alexa fluor 488-conjugated anti-rat IgG secondary antibody at a dilution of 1:200 for 60 min at room temperature. Cell nuclei were labelled by DAPI (Sigma-Aldrich). Slides were mounted with water based mounting medium and covered with a coverslip. Five images were captured from randomly chosen microscopic field of each section at x200 total magnification by a Zeiss fluorescence microscope (Zeiss). For each image, the total tissue area and the MOMA-2 or CD31 stained positive area were measured with ImageJ software (V1.4d, NIH). Data were expressed as percentage of positive staining area per analysed area. Good intra- and inter-observer reproducibility was demonstrated with mean differences of 0.063% (95% limits of agreement: −0.471–0.346; n = 12) and 0.075% (95% limits of agreement: −0.926–1.080; n = 12), respectively.

### Statistical analysis

D’Agostino Pearson test was used to test the distribution of all data. Normally distributed continuous data were expressed as mean ± SEM and compared between groups using Student’s *t-*test. Non-normally distributed data were expressed as median (interquartile range) and compared between groups using Mann-Whitney *U-*test. Data was analysed using GraphPad Prism 6 software (San Diego, CA, USA). Survival free from aneurysm rupture was assessed using Kaplan-Meier analysis and compared between groups using log-rank test. Pearson correlation coefficient test was used to assess the relationship between pro-inflammatory cytokine MCP-1 concentrations in aortic tissue and maximum SRA aortic diameters. A value of *p* < 0.05 was considered statistical significant.

## Additional Information

**How to cite this article**: Yu, H. *et al*. Angiopoietin-2 attenuates angiotensin II-induced aortic aneurysm and atherosclerosis in apolipoprotein E-deficient mice. *Sci. Rep.* **6**, 35190; doi: 10.1038/srep35190 (2016).

## Supplementary Material

Supplementary Information

## Figures and Tables

**Table 1:** Visual classification of aortic aneurysms formed in response to AngII.

Group	n	Type 0	Type I	Type II	Type III	Type IV
Control	23	3	5	0	5	10
rAngpt2	24	5	14	1	0	4

Type 0, no dilatation; Type I, dilatation of the aorta with no thrombus; Type II, dilatation of the aorta containing thrombus; Type III, a pronounced bulbous form of Type II; Type IV, multiple, complex form of Type III.

**Table 2:** Blood pressure and plasma lipids in AngII-infused ApoE^−/−^ mice at baseline (Day 0) and 14-days post administration of rAngpt2 or control.

		Day	Control (n = 7)	rAngpt2 (n = 10)	P
BP (mmHg)	SBP	0	97 (93–101)	100 (94–104)	0.361
		14	132 (98–163)*	123 (111–134)*	0.706
	DBP	0	76 (74–80)	80 (76–85)	0.108
		14	105 (79–137)*	96 (85–122)*	0.690
	MBP	0	83 (80–86)	88 (79–91)	0.184
		14	114 (85–145)*	104 (94–132)*	0.706
Cholesterol (μg/μl)	Total	0	4.4 (3.9–5.8)	3.9 (3.5–4.9)	0.297
		14	4.3 (3.2–4.6)	3.4 (2.9–4.0)	0.229
	HDL fraction	0	2.4 (2.1–3.1)	2.6 (1.3–3.6)	0.600
		14	2.5 (1.7–3.0)	1.9 (1.3–2.4)	0.133
	LDL/VLDL fraction	0	2.1 (1.3–2.9)	2.0 (1.3–2.4)	0.402
		14	1.1 (1.5–1.7)	1.2 (1.6–2.1)	0.516

Data expressed as median (interquartile range); rAngpt2, recombinant angiopoietin 2; P, p-value for comparison between groups by Mann-Whitney U test;^*^P < 0.05 compared to Day 0; BP, blood pressure; SBP, systolic blood pressure; DBP, diastolic blood pressure; MBP, mean blood pressure; HDL, high density lipoprotein; LDL, low density lipoprotein; VLDL, very low density lipoprotein.
