# Supplementary material for: Angiopoietin-2 attenuates angiotensin II-induced aortic aneurysm and atherosclerosis in apolipoprotein E-deficient mice
Source: Sci Rep. 2016 Oct 21;6:35190. doi: 10.1038/srep35190 (PMC5073347; doi:10.1038/srep35190)
Supplement: Supplementary Information [file srep35190-s1.pdf]

***SUPPLEMENTAL MATERIAL***

**Angiopoietin-2 attenuates angiotensin II-induced aortic aneurysm and atherosclerosis in apolipoprotein E-deficient mice.**

Hongyou YU, Corey S. Moran, Alexandra F. Trollope, Lynn Woodward, Robert Kinobe, Catherine M. Rush, Jonathan Golledge.

__Supplementary Figures__

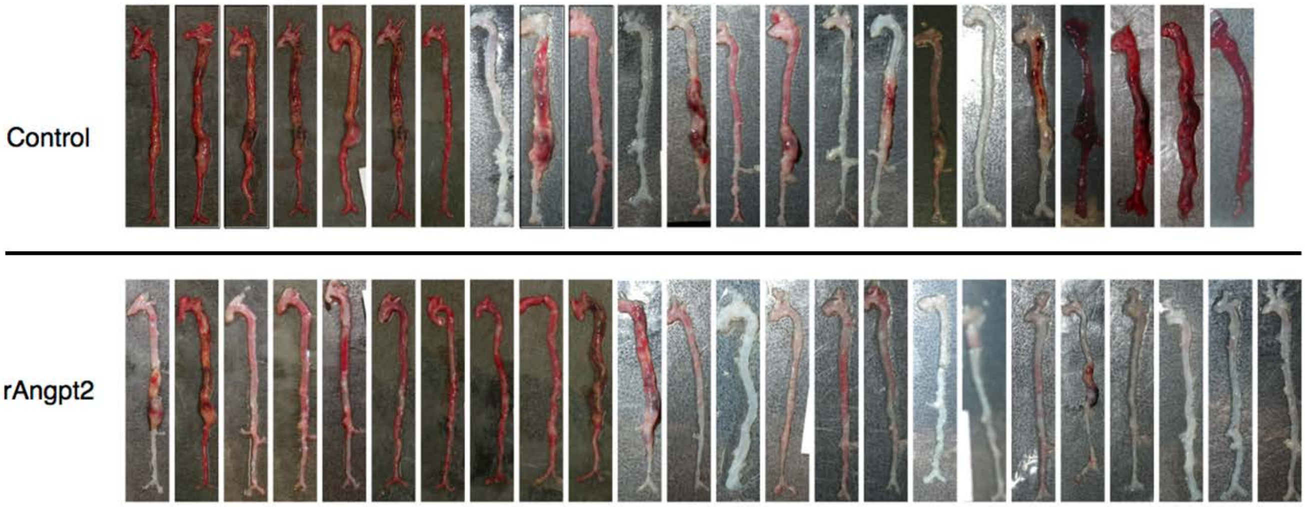


**Fig. S1:** Whole aortas harvested from AngII infused ApoE$^{-/-}$ mice that received control peptide (n=23) or rAngpt2 (n=24).

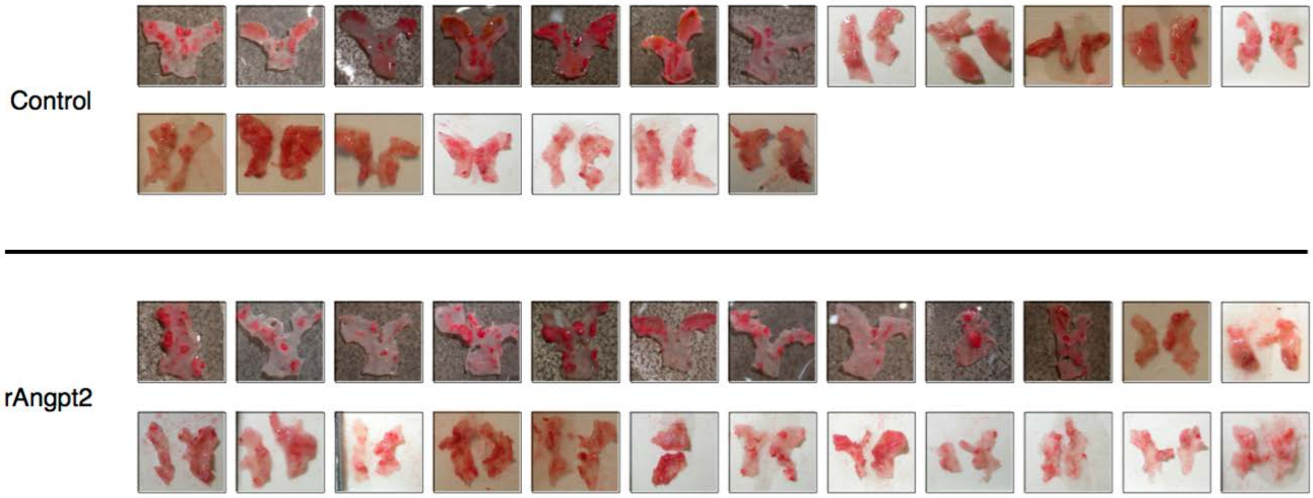


**Fig. S2:** Sudan IV staining of aortic arches from AngII infused ApoE$^{-/-}$ mice that received control peptide (n=19) or rAngpt2 (n=24).

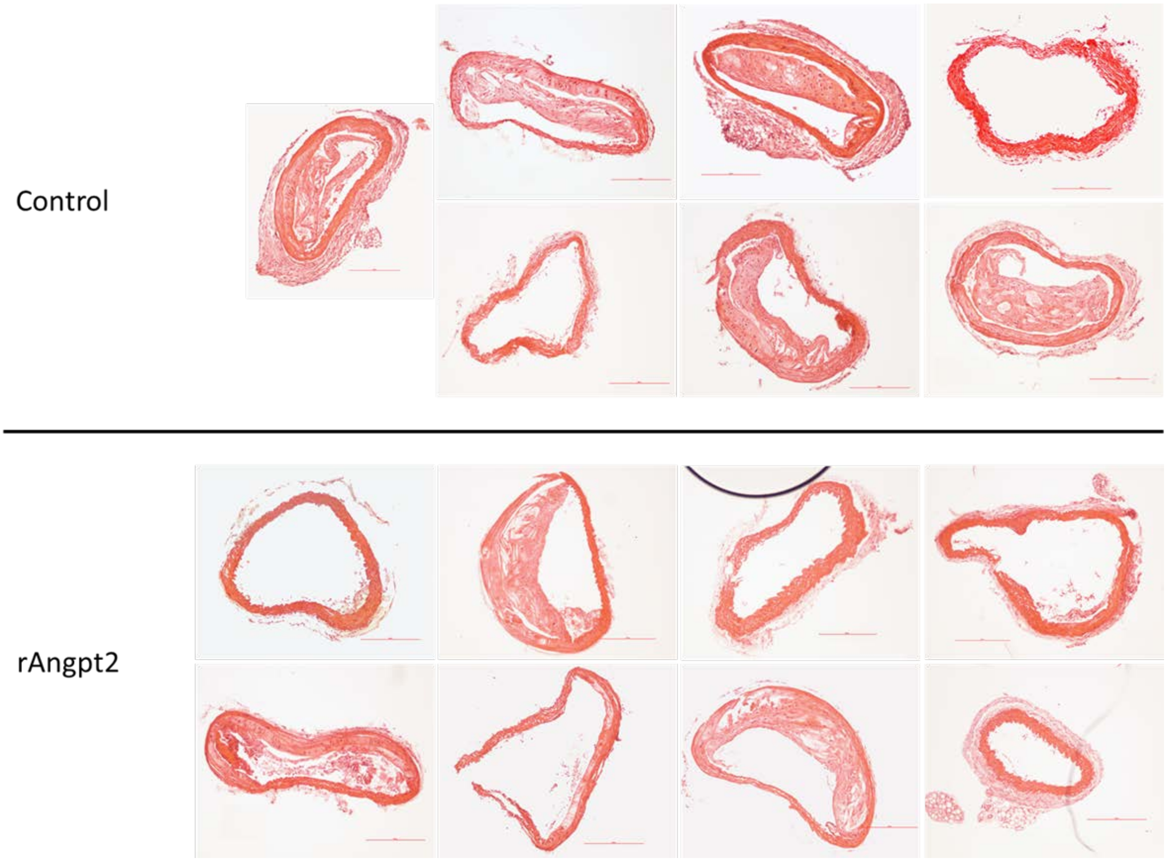


**Fig. S3:** Atherosclerotic plaque within the brachiocephalic artery from AngII infused ApoE$^{-/-}$ mice that received control peptide (n=7) or rAngpt2 (n=8). *Scale bar, 500 μm.*

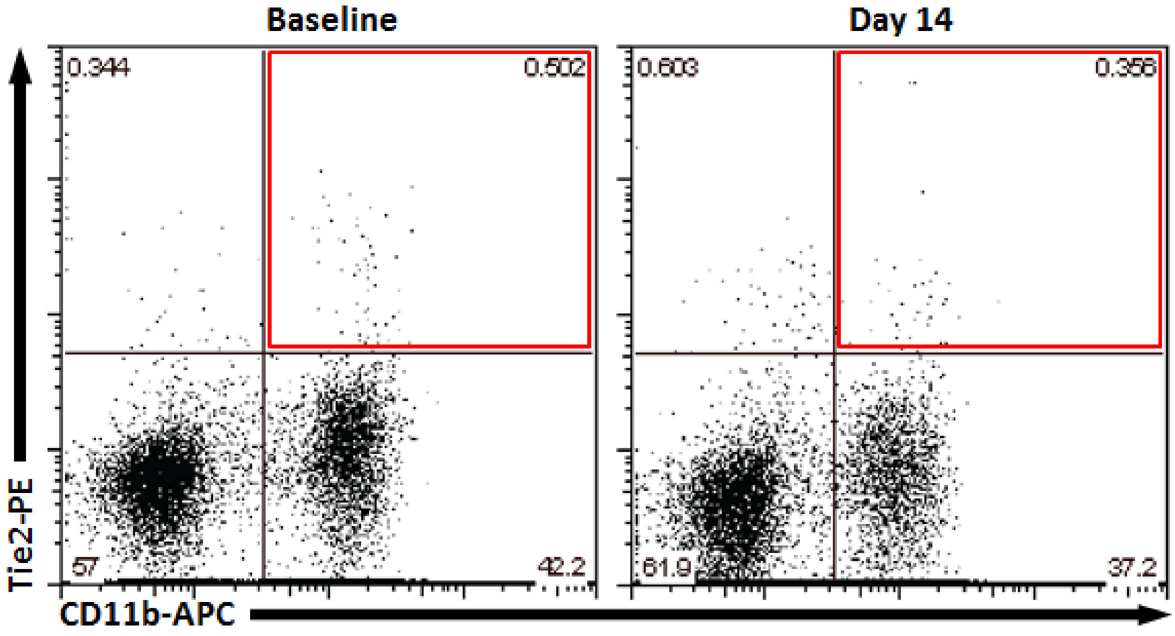


**Fig. S4:** Representative FACS plots for CD11b$^{+}$Tie2$^{+}$ leukocytes as % total (CD11b$^{+}$) population prior to (baseline) and in response to 14 days of AngII infusion.
